# scGPS: Determining Cell States and Global Fate Potential of Subpopulations

**DOI:** 10.3389/fgene.2021.666771

**Published:** 2021-07-19

**Authors:** Michael Thompson, Maika Matsumoto, Tianqi Ma, Anne Senabouth, Nathan J. Palpant, Joseph E. Powell, Quan Nguyen

**Affiliations:** ^1^Institute for Molecular Bioscience, University of Queensland, Brisbane, QLD, Australia; ^2^Garvan-Weizmann Centre for Cellular Genomics, Garvan Institute of Medical Research, Sydney, NSW, Australia; ^3^UNSW Cellular Genomics Futures Institute, University of New South Wales, Sydney, NSW, Australia

**Keywords:** single cell, machine learning, clustering, trajectory analysis, cell fate

## Abstract

Finding cell states and their transcriptional relatedness is a main outcome from analysing single-cell data. In developmental biology, determining whether cells are related in a differentiation lineage remains a major challenge. A seamless analysis pipeline from cell clustering to estimating the probability of transitions between cell clusters is lacking. Here, we present Single Cell Global fate Potential of Subpopulations (*scGPS*) to characterise transcriptional relationship between cell states. *scGPS* decomposes mixed cell populations in one or more samples into clusters (*SCORE* algorithm) and estimates pairwise transitioning potential (*scGPS* algorithm) of any pair of clusters. *SCORE* allows for the assessment and selection of stable clustering results, a major challenge in clustering analysis. *scGPS* implements a novel approach, with machine learning classification, to flexibly construct trajectory connections between clusters. *scGPS* also has a feature selection functionality by network and modelling approaches to find biological processes and driver genes that connect cell populations. We applied *scGPS* in diverse developmental contexts and show superior results compared to a range of clustering and trajectory analysis methods. *scGPS* is able to identify the dynamics of cellular plasticity in a user-friendly workflow, that is fast and memory efficient. scGPS is implemented in *R* with optimised functions using *C++* and is publicly available in Bioconductor.

## 1. Introduction

Single-cell RNA sequencing technologies (scRNAseq), enable researchers to profile the transcriptomes of thousands of cells at an individual cell resolution. One of the most important objectives of single-cell analysis is to disentangle the cellular complexity of a biological sample, especially regarding the subpopulation composition and their relationship (Lahnemann et al., 2020). Clustering analysis is commonly performed as an early analytical step to decompose cells in a sample into groups of cells with similar transcriptional profiles, often each representing a discrete cell type. Clustering, as a machine learning algorithm, can be generally categorised into supervised and unsupervised learning. Supervised learning uses reference data with labelled cells as the guidance to cluster cells and assign cell identities (Ranjan et al., 2021). In contrast, unsupervised learning partitions cells based on the transcriptional similarity between the cells (Kiselev et al., 2019). Unsupervised methods are advantageous because they offer a data-driven and unbiased approach that can be applied to any data and are useful for identifying novel cell types (Kiselev et al., 2019). Unsupervised methods are used to determine the subpopulations, while supervised methods are applied to infer the cell identity (i.e., cluster annotation). So far, there have been over 200 clustering tools developed for single-cell transcriptomic analysis (Zappia et al., 2018), each of which comes with different clustering algorithms. *k*-means and hierarchical clustering are the conventional algorithms used for clustering analysis (Petegrosso et al., 2020). Graph-based methods are another popular approach, which have been applied to some widely used software pipelines such as *Seurat* (Stuart et al., 2019) and *Scanpy* (Wolf et al., 2018).

One of the challenges of unsupervised-clustering is to define subpopulations that are robust to both technical noise and/or biological stochasticity (Kanter et al., 2019; Lahnemann et al., 2020) and parameter settings (Krzak et al., 2019). Slight gene expression variation may change the assignment of cells to different groups (Kanter et al., 2019). Most clustering tools provide multiple parameter settings, and often the number of clusters is determined by the user in an *ad hoc* manner (Lahnemann et al., 2020). A recent review identified that the performance of these tools is strongly dependent on the user-specified parameter setting as well as the dimensionality and composition of the datasets (Krzak et al., 2019). Therefore, a clustering algorithm that statistically selects clustering results most robust to parameter settings can simplify and reduce the subjectivity involved in defining subpopulation composition. Statistically justified clustering results will then need to be validated by biological experiments. In this work, we present *SCORE* as a user-friendly, statistically-tractable, and unsupervised clustering algorithm to automatically assess and select for a stable clustering result, via dynamic scanning of different clustering resolutions, followed by bootstrapping and bagging analysis.

Clustering analysis is used to find discrete cell types, often followed by downstream analyses to investigate cellular processes between cells within and among clusters. Trajectory inference constructs predicted developmental pathways of cells by ordering them along a transcriptional trajectory in cellular space (Trapnell, 2015; Cannoodt et al., 2016). Many of the currently available trajectory inference tools apply a graph-based approach (Saelens et al., 2019). Based on gene expression, cells can be arranged into a connected structure as a graph. In a graph, nodes represent cells and edges represent the pairwise expression similarity (connectedness) between cells (Wagner and Klein, 2020). Cells can also be arranged in a low dimensional manifold, where transcriptionally related cells are closely positioned in the reduced space (Trapnell et al., 2014). Principal graph algorithms can be used for such purposes (Qiu et al., 2017). Minimum spanning tree is another commonly used algorithm applied to determine the cell- and/or cluster-level trajectory (Shin et al., 2015; Street et al., 2018). These graph-based methods depend on the assumption that cells with similar transcriptional profiles will be found in proximity within a trajectory (Baron and van Oudenaarden, 2019). However, the global transcriptional similarity between cells may be biased by a range of factors, such as by highly expressed genes not specifically associated with cell commitment or by biological processes that have strong continuous expression pattern (e.g., cell cycle), which may mask differentiation processes represented in the data set (Tritschler et al., 2019). The performance of graph-based methods is also dependent on the dataset. They perform best when there is a strong continuous flux of transcriptional states in the dataset, but become less compatible and are prone to creating artefact connections when there are a small number of cells representing a biological timepoint, or when the transitional timepoints are missing from the datasets (Wagner and Klein, 2020). We developed *scGPS*, a novel algorithm that is not dependent on a range of common assumptions applied by existing trajectory methods, such as: the continuum transition between cells, the tree-like structure or predefined topology of the global lineages, and the use of all cells in a dataset to initialize a graph prior to optimisation. This allows for the flexibility to apply *scGPS* on one or multiple datasets, even with datasets generated by different studies without the need to assume any connections. This is different from existing trajectory inference methods, which assume cell clusters follow an expected topology (Saelens et al., 2019).

Although it is a common need to perform clustering and trajectory inference for a single-cell dataset, very few tools provide a streamlined pipeline to perform both analyses. Combining these two analyses from separate tools can often be problematic due to different data pre-processing pipelines that are required for each analysis type. For examples, normalisation and dimensionality reduction methods prior to clustering or trajectory analysis are diverse and can significantly affect the downstream analysis (Townes et al., 2019). Here we present *scGPS*, a software package that streamlines two advanced machine learning methods, *SCORE* clustering and *scGPS* trajectory analysis. These two algorithms enable users to assess and find stable clustering results and to predict relationships between clusters. The flexibility of the two algorithms allows for analyses of different biological contexts, especially in differentiation and cellular plasticity. Fast computation also enables the analysis of big datasets.

## 2. Methods

**Figure 1:**
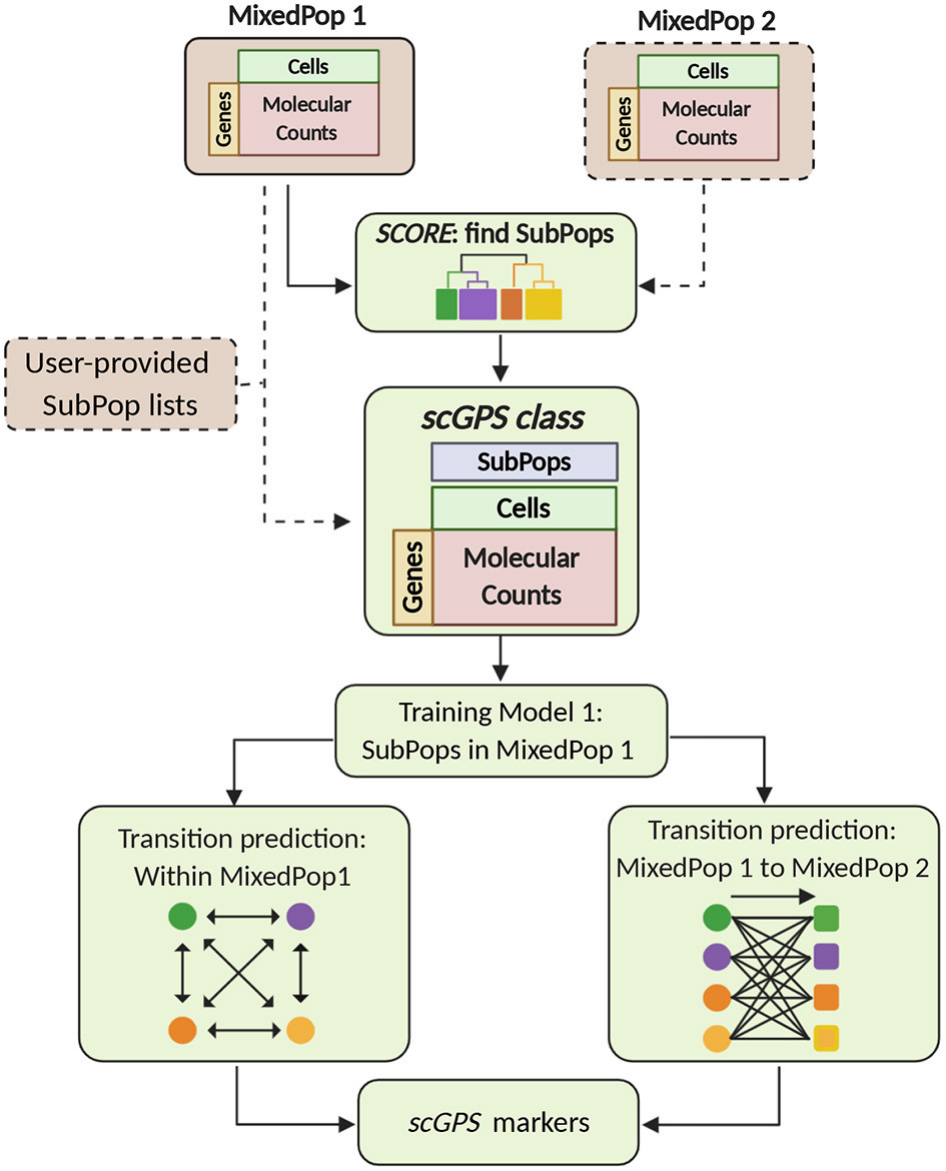
*scGPS* workflow. Brown boxes represent inputs and green boxes show the main *scGPS* analysis. The dotted boxes represent optional inputs. The input for *scGPS* analysis can be either a single expression matrix or two expression matrices of two different cell populations, the following (shown as MixedPop 1 and MixedPop 2). *scGPS* provides the functionality to determine stable clusters within a cell population by *SCORE* algorithm. Alternatively, users can provide their predetermined subpopulation list (clusters). The *scGPS* class object is based on the widely adoptable SingleCellExperiment class (Lun and Risso, 2019). Based on the subpopulation input, *scGPS* performs gene feature selection by training an Elastic net regularisation. Informative genes are then used in a logistic regression classifier to predict cell transition probability between subpopulations. When the input is one mixed sample (MixedPop1), *scGPS* computes the transition scores between different subpopulations within the same sample. When the inputs are two mixed samples (MixedPop1 and MixedPop2), *scGPS* computes the transition scores from the subpopulations in population one to those in population two. *scGPS* also predicts marker genes and their contribution to the transition between two subpopulations (refer to Supplementary Figure 1).

The two key algorithms in the *scGPS* software workflow, *SCORE* and *scGPS*, are described in Figure 1. The inputs are flexible, containing either one or more scRNAseq datasets, with or without clustering (subpopulation) information. *scGPS* can perform an end-to-end analysis from raw data to clustering, trajectory inference, gene marker selection, and visualisation. scGPS accepts either raw count or normalised count data. Since normalisation is diversely dependent on the complexity of the datatypes and each experimental design, users would often perform normalisation using their own methods of choice, for example, cell-to-cell normalisation can be done by Scran or scTranform (Seurat). For differential expression (DE) analysis in scGPS, raw data or rounded normalised data are processed by a wrapper function of DEseq2 (Love et al., 2014) through fitting dispersion-mean relationship across samples/cells. DE analysis is not the focus of scGPS, as it is used to obtain a gene list to initialize the feature selection step through ElasticNet regularisation procedure. The gene list can be provided by users as an input for scGPS, in which case DE analysis is not required for the scGPS workflow. The gene marker selection and visualisation steps are shown in the Supplementary Figure 1. Detailed descriptions of the two main algorithms in scGPS are presented below.

### 2.1. SCORE Clustering Algorithm

*SCORE* is an unsupervised clustering algorithm, an extension on previous work *CORE* (Nguyen et al., 2018; Senabouth et al., 2019), with additional stability analyses. The method is the first step in the *scGPS* package. Similar to *CORE* as described in Nguyen et al. (2018) and Senabouth et al. (2019), clustering in *SCORE* starts by building a hierarchical distance tree between cells. *SCORE* uses Wishart's version of the Ward algorithm, implementing the Lance-Williams update formula to find an optimal grouping of branches to make stable clustering results (Lance and Williams, 1967; Wishart, 1969). Expanding on the *CORE* algorithm (Nguyen et al., 2018; Senabouth et al., 2019), *SCORE* adds bagging strategies to search for a stable clustering result. From the original Euclidean distance matrix calculated when building the dendrogram of the whole dataset as applied in *CORE*, here *SCORE* subsamples a proportion of the matrix.

This method quickly generates dendrogram trees of variable sizes without the need to recalculate cell distances, which typically is the most computationally expensive step of clustering. We select the optimal cluster resolution by implementing tree-height iterations and bagging strategies according to **Algorithm 1** below.

**Algorithm 1:** *SCORE* Algorithm

1 Create a dendrogram tree using *CORE*, keeping Euclidean distance matrix *M* for bagging runs:
2 Create a vector b_*k*_ (*k* = 1, 2, …, *m*);
3 Populate b_*k*_ with a subsample of cells, with replacement, from the set of all cells C;
4 Create a new matrix, *N*_*k*_, of Euclidean distances for the cells in b_*k*_, using values from *M*;
5 Generate a new dendrogram tree and clustering of cells;
6 Record result from optimal stability of subsampled tree;
7 Vote on most commonly occurring result;
8 Choose most stable result from the original dendrogram tree

### 2.2. The *scGPS* Prediction Algorithm

We developed an unsupervised machine learning approach to predict differentiation trajectories between any two sub-populations (two clusters) within one dataset or between two independent datasets. This approach does not rely on assumptions that define many current methods, including: (1) the trajectory needs to be continuous between the two sub-populations, (2) the trajectory follows a defined topology, (3) the trajectory is unidirectional, (4) data of all cells are needed to initialise the algorithm (refer to Tritschler et al., 2019).

After obtaining the clustering information, by *SCORE's* algorithm as described above (or by an independent clustering algorithm if the data input does not require clustering), we can estimate the relatedness between any two clusters within or between datasets. Based on gene expression data, we calculate the class probability of a cell belonging to a sub-population or not. For every subpopulation, we find the number of cells that can be in the same class with the target subpopulation, meaning to be more transcriptionally related than compared to other subpopulations (classes). *scGPS* calculates the proportion of cells in one subpopulation with the binary conditional class probability that the cell belongs to a targeted class (targeted subpopulation). This proportion is defined as the transitioning potential between the two subpopulations, with 0 being unlikely transition and 1 being the most likely. Notably, the transitioning is directional, allowing for the estimation of a transition probability from cluster 1 to cluster 2 and a probability from cluster 2 to cluster 1. In *scGPS* workflow, within each given dataset, a sub-population is distinguished from the remaining cells in the dataset by a Least Absolute Shrinkage and Selection Operators (LASSO) (Tibshirani, 1996) and cross-validation procedure. The LASSO model training for the dataset selects the most predictive genes that distinguish the cells in the subpopulation from all the remaining cells in that dataset (Supplementary Figure 1). LASSO-selected genes are then considered as the gene features for the subpopulation, and these genes will be used for *scGPS* prediction (**Algorithm 2**, Equation 1). As a result of the model training, those genes not informative for classifying the cells in the subpopulation have coefficients reduced to 0. The remaining genes with coefficients bigger than 0 are those that are predictive of the transition between clusters, and thus can be considered as trajectory driver genes. These genes can be visualised in scGPS as shown in Supplementary Figure 1B. The cluster with the LASSO-defined genes is considered a source cluster, in which the expression of each cell will be fitted into the logistic classifier (Equation 2) to compute the probability that the cell belongs to the same class of the target subpopulation to be compared to or not. The probability suggests the transcriptional relateness to the target subpopulation relative to other subpopulations in the original dataset where the source subpopulation is defined. The target subpopulation can be in the same or in different datasets. This way, *scGPS* allows for the comparison of any pair of subpopulations, as described in **Algorithm 2**.

**Algorithm 2:**
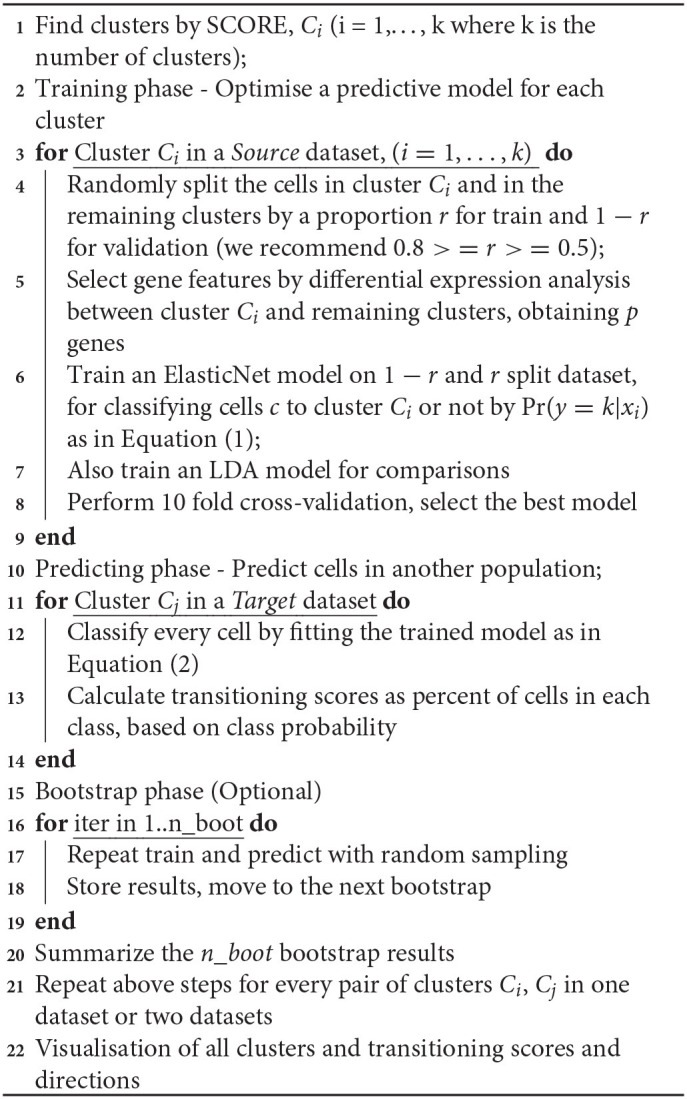
scGPS trajectory analysis

Let subpopulation labels be a categorical response variable *y* and assign *y* into one of two classes, belonging or not belonging to cluster *C*_*i*_. Let *p* equal the number of gene predictors. For each subpopulation, we fit a generalised linear model (binomial distribution) with the response variable as a vector containing two classes (∈*C*_*i*_ and ∉*C*_*i*_), and the predictor as the matrix *n* cells by *p* genes of the expression levels for the classes cells. Effect sizes β_*j*_ of the genes *x*_*j*_ are estimated by a penalised maximum likelihood procedure. The resulting model with the optimal set of non-zero coefficient genes is a Bayes optimal classifier. The model removed insignificant genes that do not contribute to the model fit by shrinking their coefficients to 0 following:

(1) $$\begin{array}{l} \text{argmin}\left(1/\text{N}\overset{\text{n}}{\underset{\text{i}=1}{\sum }}\text{l}\left({\text{y}}_{\text{i}},\beta_{0}+\overset{\text{p}}{\underset{\text{j}=1}{\sum }}\beta_{\text{j}}{\text{x}}_{\text{ij}}\right)+\lambda \overset{\text{p}}{\underset{\text{j}=1}{\sum }}|\beta_{\text{j}}|\right) \end{array}$$

where *x*_*i*_ = (*x*_*i*1_, *x*_*i*2_, …, *x*_*ip*_) is a vector of expression values of p genes in cell *c*_*i*_; *y*_*i*_ is the cell subpopulation label of the cell *c*_*i*_; $l\left(y_{i},\beta_{0}+\overset{p}{\underset{j=1}{\sum }}\beta_{j}x_{ij}\right)$ is the negative log-likelihood for *C*_*i*_; and λ is a tuning parameter that controls the shrinkage penalty of the coefficients. For each training cell subpopulation, an optimal λ and a set of gene predictors can be determined by a 10-fold cross-validation procedure to select the λ that produced the minimum classification errors. The LASSO procedure optimizes the combination set of coefficients for all predictors in a way that the residual sum of squares is smallest for a given λ value.

The conditional class probabilities of cell *c* belonging to *C*_*i*_ is the linear combination of selected genes can be used to classify every cell:

(2) $$\begin{array}{l} ln\left(Pr\left(y=1|X=x\right)\right)=\beta_{0}+\beta_{1}x_{1}+\beta_{2}x_{2}+\ldots +\beta_{p}x_{p}=\beta_{0}+x\beta \end{array}$$

where β_*j*_ is a coefficient for gene *j* (β_*j*_ = 0 if the gene *j* is not a predictor in the class). The coefficient vector β = (β_0_, β_1_, β_2_, …, β_*p*_) is calculated by maximum likelihood estimation. The predicted probability of a cell *c* being in a subpopulation *C*_*i*_ or $\bar{C_{i}}$ is estimated by replacing β and gene expression values to the regression equation. scGPS transition score can be related to transition probability in Markov chain in that two clusters are connected by conditional probability. The main differences are that scGPS works with any two clusters without an assumption on the time sequence or order of these clusters in the trajectory and scGPS estimates class probability for every cell, independently of other cells.

Importantly, for stable results, *scGPS* has an option to run *n* bootstraps to allow averaging of the percent of transitional cells from one sub-population to another. In addition, as a control for LASSO, we also include an LDA classifier (Linear Discrimination Analysis), allowing for comparisons between a full and a shrunken model.

## 3. Results

### 3.1. Stable Clustering

**Figure 2:**
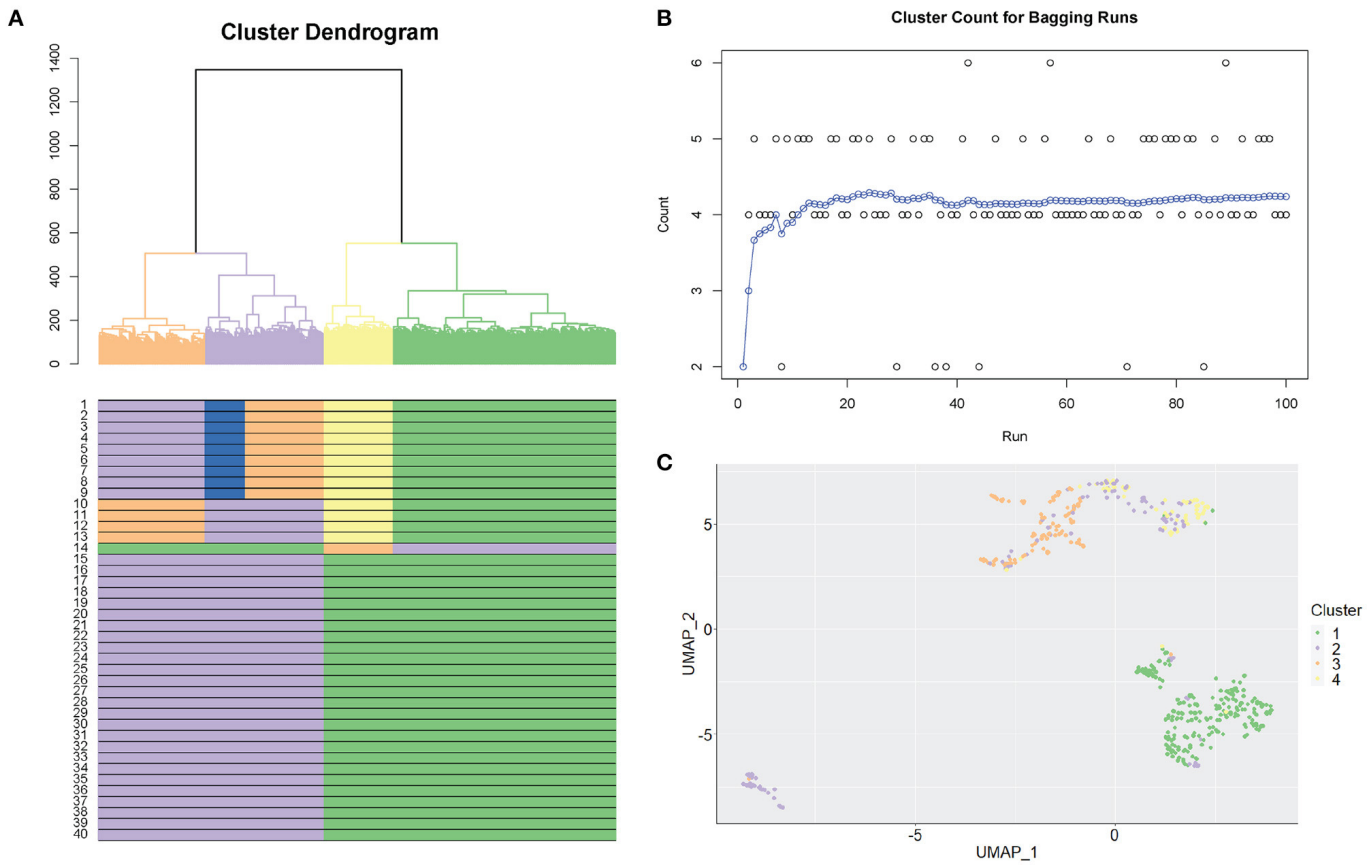
Selection of stable clustering results. **(A)** *SCORE* cluster dendrogram. Coloured branches and bars (underneath the dendrogram) represent clusters. A coloured row displays the result from one clustering resolution (40 resolutions are shown). **(B)** Bagging cluster estimations. Each dot is one bootstrap result. The X-axis shows the order of the bootstraps from 1 to 100. The y-axis shows the number of clusters. Blue dots are the cumulative running average between consecutive clusters (i.e., continuous bootstraps). **(C)** UMAP plot representing the final chosen clustering result. Each colour represents one cluster.

Figure 2 shows an example of the selection of clusters using the *SCORE* algorithm. We used the Smart-seq human cerebral organoids by Camp et al. (2015), with processed data from the Hemberg collection (https://hemberg-lab.github.io/scRNA.seq.datasets) that had been reduced to 553 cells with reference cluster labels. Figure 2A illustrates the original cluster dendrogram, with 40 windows underneath the dendrogram, and their corresponding clustering results for the original data. In this example, the process of dendrogram generation is repeated 100 times with different subsampled populations. Figure 2B shows the optimal number of clusters found for each new tree formed. This panel also tracks the moving average of the bagging runs to gauge the stability of the method. From the information supplied, the clustering result containing four clusters is chosen. The chosen clustering result is illustrated in the dendrogram in Figure 2A, with matching results seen in the windows below. We can also discover additional information about the population in Figure 2B, where we examine the individual results from the bagging runs. Bootstrap results are distributed on mainly four clusters and also regularly five clusters, indicating the stability at those resolutions. Notably, there were several times a sixth cluster, not present in the original groupings, was found as the results from the random bootstrapping procedure. This is a further step to examine the possibility of smaller clusters that otherwise would have been masked by larger subpopulations if it was not for subsampling. Visual representation of clustering changes across 40 windows using different biological datasets is shown in Supplementary Figure 2. The clusters in these datasets range from simple (3) to more complex (7) and from hundreds to thousands of cells.

### 3.2. Trajectory Analysis

**Figure 3:**
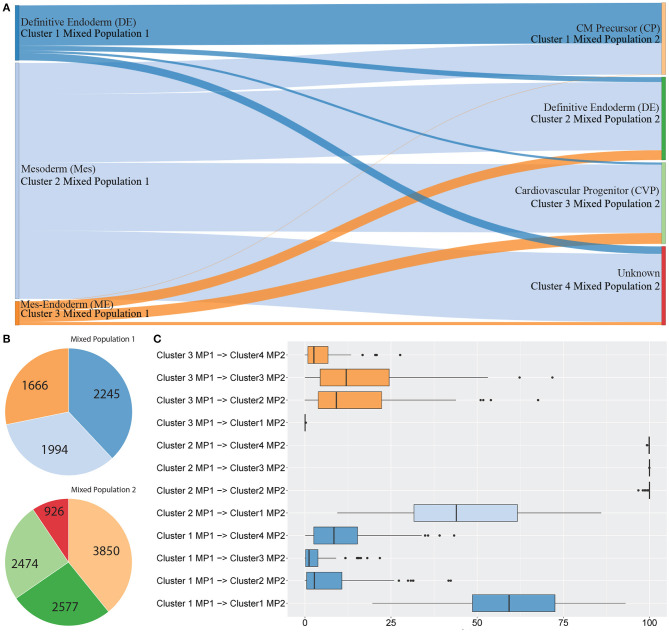
*scGPS* trajectory analysis. *scGPS* trajectory analysis can perform transitioning prediction between two populations (this Figure 3) or within a heterogenous population (Figure 4). In **(A)** edges show pairwise connections between two clusters, where each node (a vertical bar) represents a cluster, and the edge width is proportional to the transitioning score from one cluster to another. MP represents a mixed population (i.e., the total dataset for one mixed sample, containing multiple clusters). Each coloured bar represents one subpopulation (a cluster). **(A)** Transitioning between three subpopulations in the mixed population 1 (MP1) to four subpopulations in the mixed population 2 (MP2). **(B)** The number of cells in each cluster shown in **(A)**. **(C)** Bootstrapping results displaying the summary scores from one hundred bootstraps used for the same data shown in **(A)**.

**Figure 4:**
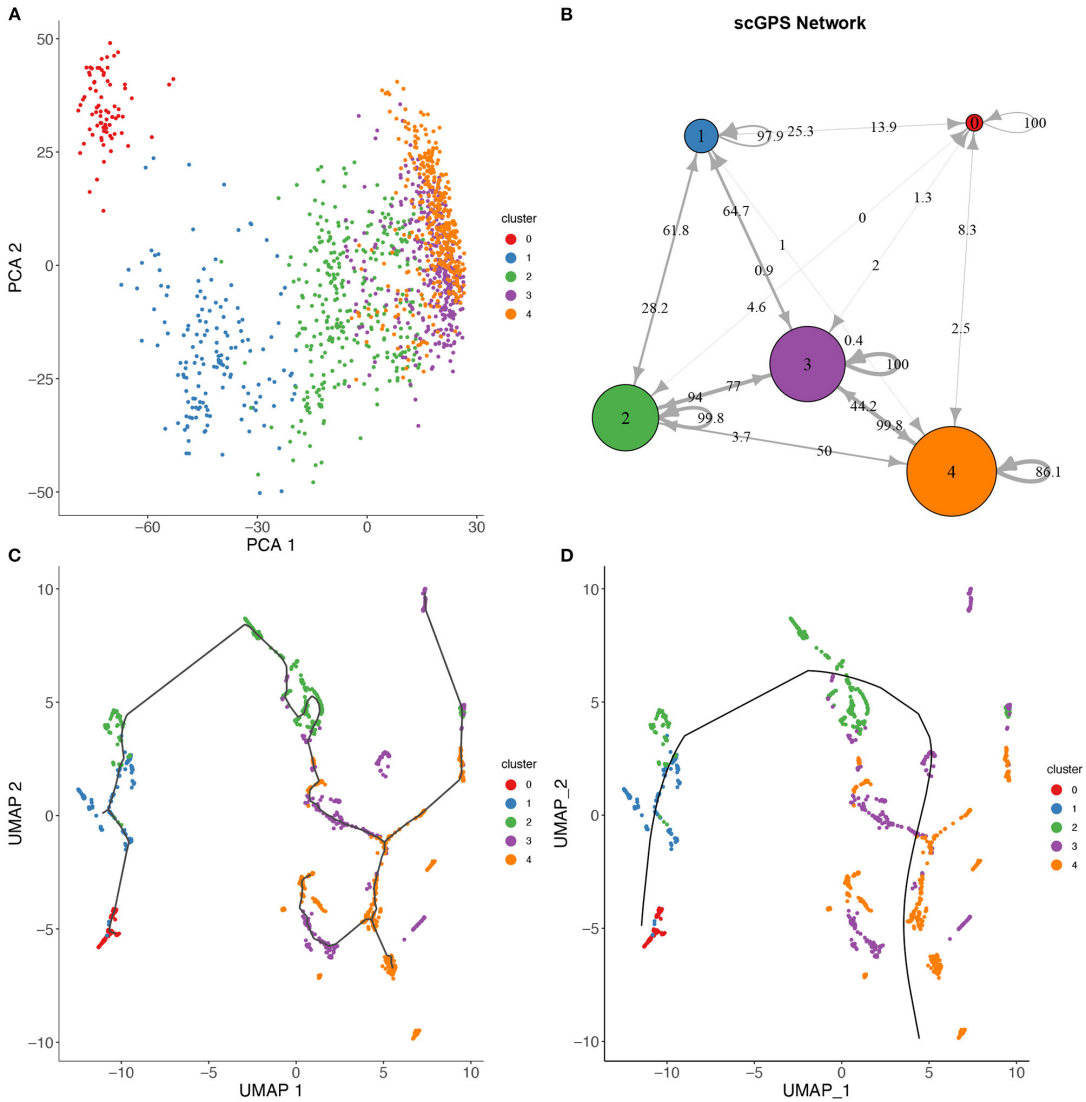
scGPS trajectory comparisons with methods Slingshot and Monocole on the full-length total RNA sequencing dataset from Petropoulos et al. (2016), processed by Saelens et al. (2019). **(A)** PCA dimensionality reduction of the dataset. The data is labelled with timing information for the cells which were collected at 5 time points. **(B)** *scGPS* trajectory analysis. Arrows show direction and numbers show transitioning scores. For example, number 50 on the arrow from cluster 2–4 indicates a score of 50% total cells transitioning from 2 to 4. **(C)** Monocole trajectory graph. **(D)** Slingshot trajectory graph. The black, smoothed curve shows predicted linage, connecting clusters represented by coloured dots.

Figure 3 shows between population analysis (Figures 3A,C). Here we analysed selected data from a time-course dataset capturing differentiation of induced pluripotent stem cells (iPSCs) into cardiomyocytes (Friedman et al., 2018). These data represent cell transitions that follow classical developmental lineage decisions through mesendoderm cell types into differentiated cell types. Time-course datasets like this that involve major cell state changes between cell captures are particularly challenging to analyse by trajectory prediction because significant transcriptional changes make cell transitions difficult to predict. We used *scGPS* to investigate the transition of cell types from three clusters in day 2 (representing gastrulation-stage mesendoderm cell types) to four clusters in day 5 (representing progenitor cells including definitive endoderm, endothelium, and cardiomyocyte precursor cells). The biological annotation for each cluster and the transitioning between cell types were described in their original paper (Friedman et al., 2018). Using the same input data with the data in the original paper, *scGPS* predicts the transitioning probabilities between every pair of clusters and offers three visualisation options for analysing output predictions (Figures 3, 4B, and Supplementary Figure 3). Consistent with the result reported (Friedman et al., 2018), *scGPS* found cells in cluster 2 (mesoderm) were predicted to transition into four cell types (cardiovascular precursor/progenitor and definitive endoderm) in the day 5 dataset (Figures 3A,C) (Friedman et al., 2018). On the other hand, cluster 3 (mesendoderm) and cluster 1 (endoderm) were not predicted to progress into day 5, which is consistent with reported results and also follows with expected mesendoderm cell differentiation lineage relationships (Figures 3A,C). Figure 3B shows cell numbers in each of the clusters, with the cluster colours matching the colours of corresponding clusters shown in A and C. *scGPS* analysis also assesses the consistency of the prediction through a bootstrap run, where only a subset of randomly sampled cells are used. Figure 3C shows the results from 100 runs, suggesting a high level of confidence for the prediction from cluster 2 (mesoderm) on day 2–5 but not cluster 1 (definitive endoderm) and cluster 3 (mesendoderm). We also assessed scGPS trajectory analysis in four additional datasets, with an increased level of complexity to connect samples containing from 3 to 6 clusters (Figure 4 and Supplementary Figure 3).

### 3.3. Benchmarking of *SCORE*

To verify the processes of the SCORE clustering algorithm, the method's clustering results were benchmarked against another clustering package, *SC3* (Kiselev et al., 2017). *SC3* was chosen as it is a state of the art clustering package that also includes methods for unsupervised cluster count estimation (Kiselev et al., 2017). Table 1 outlines the benchmarking of SCORE against *SC3*. Six data sets were used of various size, data types and complexity to examine how each method performed in terms of both accuracy and speed. We used the Adjusted Rand Index (ARI) (Rand, 1971; Hubert, 1985), a widely used and adjusted for random assignments method, for quantifying the accuracy of the two methods in comparison to the clustering results reported in the original studies, across six datasets. Of note, since there was no ground-truth for the cluster label of each cell, the use of the original clustering results as the reference points for *SC3* and *scGPS* should be considered as the suitable references that are relatively accurate compared to the ground truth and those clustering results have been validated by the respective studies. Using real biological data for benchmarking rather than simulated and/or cell lines was a relevant strategy to assess the performance of the models on the real complex biological context. From our testing, both of the methods performed similarly with neither consistently outperforming each other between the datasets (Table 1). Regarding the computation time, *SCORE* outperformed the benchmark method, *SC3*, for all datasets (Table 1). For the small datasets running time difference was small but for the larger datasets SCORE well outperformed showing superior scaling. Notably, *SCORE* resulted in smaller numbers of clusters, while *SC3* found, in some cases (e.g., Baron et al., 2016), over 50 clusters compared to the 14 clusters reported in the original study.

**Table 1:** Benchmarking of clustering results and running time.

**Data set**	**Methods ARI**			**Running time**		**Number of clusters**		
	**Stable res SCORE**	**High res SCORE**	**SC3**	**SCORE**	**SC3**	**Stable res SCORE**	**High res SCORE**	**SC3**
Baron	0.613	0.613	0.265	23.934 min	137.735 min	9	9	54
Klein	0.800	0.800	0.636	3.342 min	15.891 min	6	6	16
Camp	0.559	0.597	0.556	0.544 min	2.693 min	4	5	10
Koh	0.565	0.661	0.824	0.696 min	3.239 min	7	8	18
Kumar	0.574	1.000	0.994	0.281 min	0.496 min	2	3	4
Yan	0.588	0.588	0.650	0.108 min	0.247 min	3	3	6

*Individual tests can be found through the links at: https://imb-computational-genomics-lab.github.io/scGPS/index.html. Processed data in the repository by Soneson and Robinson (2018) for the datasets generated by: Koh et al. (2016) (C1 Fludigm sequencing, 651 Human mesoderm cells, 10 Clusters) and Kumar et al. (2014) (Smart-seq, 268 mouse Embryonic stem cells, 3 Clusters). Processed data in repository from the Hemberg collection (https://hemberg-lab.github.io/scRNA.seq.datasets/) for the datasets generated by: Baron et al. (2016) (inDrop sequencing, 8569 pancreatic cells in human, 14 clusters), Klein et al. (2015) (inDrop sequencing, 2717 mouse ES cells, 4 Clusters), Camp et al. (2015) (Smart-seq, 553 human cerebral cortex organoids, 5 Clusters) and Yan et al. (2013) (Smart-seq, 90 human embryonic stem cells, 7 Clusters). Stable Res SCORE indicates the optimal resolution, most robust to parameter changes. High Res SCORE corresponds to the original number of clusters before the window-scanning procedure to search for stable clusters. The High Res SCORE corresponds to the clustering result in the first row underneath the dendrogram tree as in Figure 2A*.

### 3.4. scGPS Validation and Benchmarking

Figure 4 shows a trajectory comparison between *scGPS* and two widely adopted methods for trajectory analysis, *Slingshot* (Street et al., 2018) and *Monocole3* (Cao et al., 2019), found as top-performing among 45 methods (Saelens et al., 2019). These two methods are similar to *scGPS* in that no predefined topology assumption (e.g., linear or bifurcation) was assumed. We used a time series dataset with processes data in the repository by Saelens et al. (2019) for the dataset generated by Petropoulos et al. (2016). The data captured a transcriptional map of mouse embryo development from E3 to E7. The three methods were compared using the same cluster assignment (5 clusters, 0–4, representing the time points E3, E4, E5, E6, and E7, respectively). To allow for comparisons of trajectory analysis, we used a common UMAP dimension reduction to determine the lineages with Monocle3 and Slingshot. Figure 4A also displays a PCA reduction to visualise cells more distantly separated in the PC1 and PC2.

*scGPS* on the other hand works directly with the original gene expression space. *scGPS* trajectory inference result agrees with that of Monocole3 and Slingshot with its strongest transition scores through the path from clusters 0 → 1 → 2 → 3 → 4, consistent to the time-course of the cell development from E3 to E7. *scGPS* not only predicted the transition but also estimated the transition scores (probability of cells transitioning between clusters), which could be correlated to the transition strength. In the tested dataset, we found *scGPS* transitioning scores from 0 to 1, 1 to 2, 2 to 3 and 3 to 4 as: 25.3, 28.2, 77, and 99.8%. When the data is viewed from the perspective of each individual vertex of the network, the leaving edges, representing transitions to other clusters, maintained that the largest transitional probability was along the trajectory consistent with the timing information and the trajectories found using *Monocle3* and *Slingshot*. Transitions between other clusters were consistently lower, especially for more separated clusters along the trajectory. The transition scores give higher values between the later time points where the cells appear to be more heterogeneous, as also seen in the dimensionality reductions presented in Figures 4A,C,D. Notably, cluster 3 is indicated by PCA and UMAP plots (Figures 4A,C,D) as the most plastic cluster that is linked (mixed) with other clusters, consistent to *scGPS* prediction results. The unique feature in *scGPS* is the prediction of all possible pairs, including reverse transition, making it possible to suggest main transition and bidirectional transition, which can be particularly useful in several biological contexts.

## 4. Discussion

In this work, we introduced two main algorithms, *scGPS* and *SCORE*, to address two main single-cell analysis categories. Trajectory analysis using *scGPS* classification algorithm is novel in that the method does not use any assumption about a trajectory, for example, an assumed topology connecting cells and/or a continuous differentiation from one state to another. Different to most other trajectory inference analyses, *scGPS* does not find cell locations on a continuous, low-dimensional manifold or in a node of a graph-based trajectory, but implements a machine learning classification framework. Conditional class probability is used as the abstracted transitioning potential for a cell in one cluster to have the transcriptional potential to turn into another cluster. Most other methods, such as RNA velocity as implemented in *scVelo* (Bergen et al., 2020), are suitable for processes that happen within a narrow transitioning windows, for example differentiation within one time point. RNA velocity, however, is less suitable for cases where there are transitioning gaps between two distant samples such as two timepoints during *in vitro* differentiation of cardiomyocytes (Friedman et al., 2018).

*scGPS* is free from the need to preorder cells in local and/or global structure, either as connected manifold (like *Monocle* Cao et al., 2019) or disconnected manifold (abstracted graph approaches, as in *PAGA* Wolf et al., 2019). The abstracted graph approaches do not assume tree-like structure, but still require all data to first establish relationships between all nodes in the graph, for example to initialize a starting nearest-neighbour graph before graph optimisation (Tritschler et al., 2019). On the other hand, *scGPS* is unique in that it is free of any assumptions mentioned above. *scGPS* compares every pair of clusters, including those that are at different stages in the trajectory and based on the pairwise transitioning score, the trajectory can then be determined. Also, differentiation is not always unidirectional, but loops can happen, for example in the cases of converging/diverging behaviours (Tritschler et al., 2019). *scGPS* allows us to find such loops.Trajectory inference generates hypothetical lineages that often require biological knowledge and experiments to confirm. Therefore, we aim for the scGPS trajectories to be tractable. In the scGPS trajectory analysis, the inferred trajectory and directionality are fully explainable. The explainability is based on gene markers used as features in the classification model, how these features are selected, the defined weights of these features in the linear classifier, and the cells in the target clusters classified as in the same class as the source cluster or not. These defined parameters help with evaluating the resulting trajectory. Through benchmarking analysis, we found that scGPS inferred trajectories are consistent with the biology and results in various datasets.

The *SCORE* clustering algorithm automatically finds the number of clusters most robust to parameter changing, an important feature that most clustering methods overlook. To find consensus cluster is challenging. Our *SCORE* method focuses on finding stable clusters, robust to changing parameters by iterative bagging and bootstrapping as described above. Notably, *SCORE* does not rely on dimensionality reduction, as opposed to most other clustering methods, which perform clustering based on the reduced dimensions. Dimensionality reduction methods are variable and diverse, ranging from commonly used as PCA, tSNE, UMAP to improved variants such as CIDR or GML-PCA and to deep learning like DCA (reviewed by Sun et al., 2019). Therefore, existing clustering methods would produce variable results depending on the reduced dimension. Working on the original gene expression space is made possible in *SCORE* by implementing fast matrix computation methods, allowing the processing of large datasets with thousands of cells.

Among the over 802 tools that are available for single cell analysis, clustering (202 tools) and trajectory inference (103 tools) are the most popular analysis categories (Zappia et al., 2018). However, these two types of analyses are often not streamlined in one package. Even in widely used pipelines like *Seurat* (Stuart et al., 2019), *Scater* (McCarthy et al., 2017), and ascend (Senabouth et al., 2019), clustering is implemented, but not trajectory inference. On the other hand, popular trajectory analysis tools like *scVelo* (Bergen et al., 2020) and *Slingshot* (Street et al., 2018) do not have a clustering option. Several trajectory methods such as *Monocle3* (Cao et al., 2019) and *PAGA* (Wolf et al., 2019) focus on trajectory analyses and include a standard clustering step. Ideally a software tool that equally focuses on both clustering and trajectory analysis will be useful for broad users, especially for biologists with limited programming experience.

We expect that *scGPS* can be broadly applied in multiple contexts. Both clustering and trajectory analyses are important in deciphering the complexity of one or more sample(s). *scGPS* solves this challenging task by a streamlined analysis involving both finding clusters and comparing those clusters, either in discrete manners between cell types or in probabilistic and continuous transitions in transcriptional states (e.g., trajectory analysis). Besides implementing the two key analysis types, *scGPS* also has convenient functions to annotate clusters, find markers, and visualise clusters and their transitioning potential.

In summary, *scGPS* is a user friendly and computationally efficient software package that streamlines single cell analysis in a framework that addresses two key tasks; decomposing a mixed population into clusters and analysing the relationship between clusters (Supplementary Figure 4). The *scGPS* package offers ease of use to the user while still allowing for customisation as they require. *scGPS* holds its unique flexibility, stability and performance against the top of the line with the additional benefit of fast computational time by design, assisted by the use of C++ implementation for demanding calculations. Such features would allow users to apply scGPS for diverse usage scenarios. For example, scGPS can be applied for many granular clusters (blocks) as defined by a fine-grain partitioning algorithm like *MetaCell* (Baron et al., 2019). In this case, transition scores can be calculated between any pairs of metacells.

## 5. Software Availability

- Project name: scGPS
- Project github: https://github.com/IMB-Computational-Genomics-Lab/scGPS
- Project home page: https://imb-computational-genomics-lab.github.io/scGPS/index.html
- Bioconductor Project doi: 10.18129/B9.bioc.scGPS
- Operating system(s): platform independent
- Programming language: R and C++
- Other requirements: R(>3.6), make
- License: GPL 3.0.

## Data Availability Statement

Publicly available datasets were analyzed in this study. This data can be found here: https://zenodo.org/record/1443566#.YCNw7C2r2i6; https://hemberg-lab.github.io/scRNA.seq.datasets/ or https://github.com/hemberg-lab/scRNA.seq.datasets; http://imlspenticton.uzh.ch:3838/conquer/.

## Author Contributions

QN, JP, NP, and MT developed the concepts and algorithms. QN and MT wrote the software. QN, MT, MM, TM, NP, and JP wrote the manuscript. All authors contributed to software development and analysis and data interpretation.

## Conflict of Interest

The authors declare that the research was conducted in the absence of any commercial or financial relationships that could be construed as a potential conflict of interest.
