# Supplementary material for: scGPS: Determining Cell States and Global Fate Potential of Subpopulations
Source: Front Genet. 2021 Jul 19;12:666771. doi: 10.3389/fgene.2021.666771 (PMC8326972; doi:10.3389/fgene.2021.666771)
Supplement: Supplementary file 1 [file Data_Sheet_1.PDF]

Supplementary Material

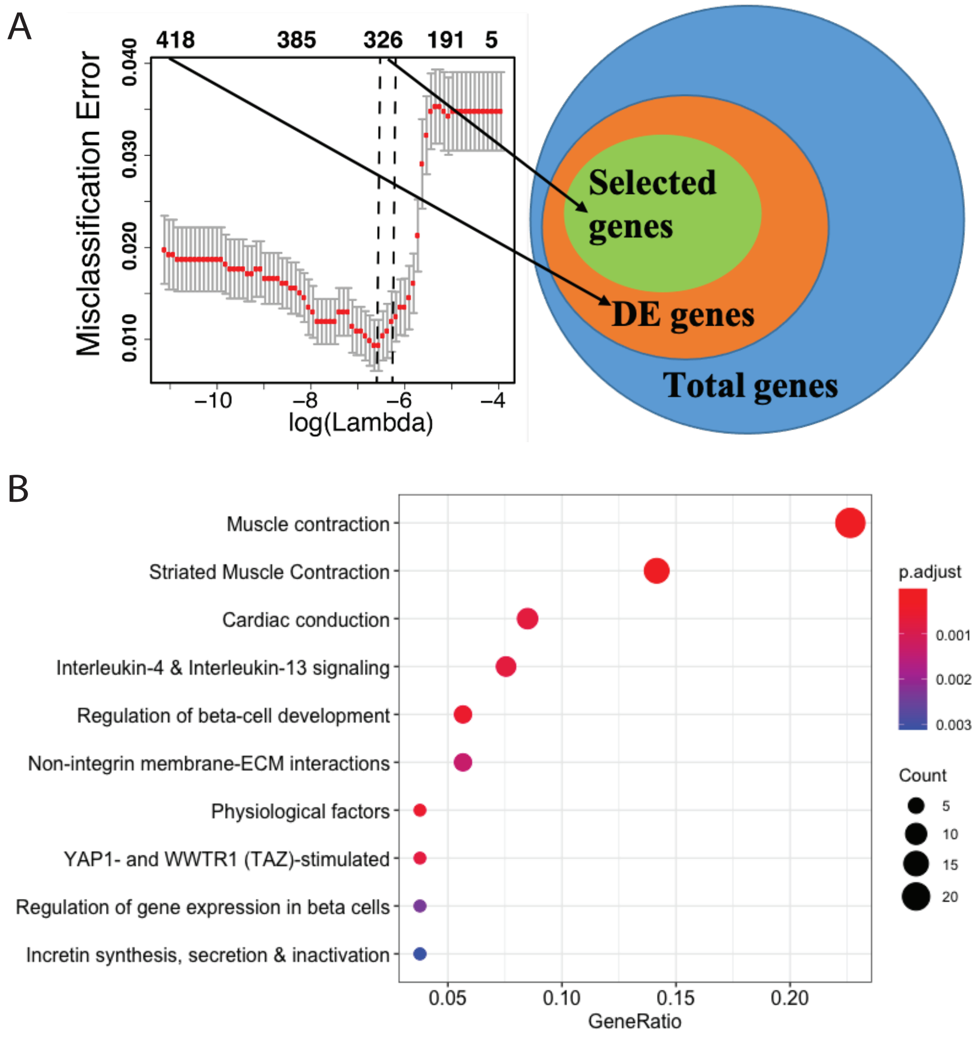

Figure S1. Gene feature selection and functional annotation. **A**, The gene selection strategy to filter from over 22,000 genes to 10-200 genes that are most predictive of a subpopulation. These genes are transcriptionally informative to distinguish the subpopulation from all the remaining cells in a dataset, and can be considered as a gene signature of the subpopulation. The feature selection process has two steps. Starting with differential gene expression analyses, we select genes that are significantly different between the cells within the subpopulation of interest compared to the remaining cells in the dataset. Next, these genes are fit into a multivariate penalised logistic regression model to classify cells belonging to or not belonging to the population of interest. During model training, genes that are not predictive will be removed (i.e. coefficent shrunken to 0). The optimised model will have the most predictive genes, with a minimal number of predictors while maintaining an optimal model performance. **B**, The selected gene features can be used in a standard pathway analysis to find biological annotations that the gene list represents.

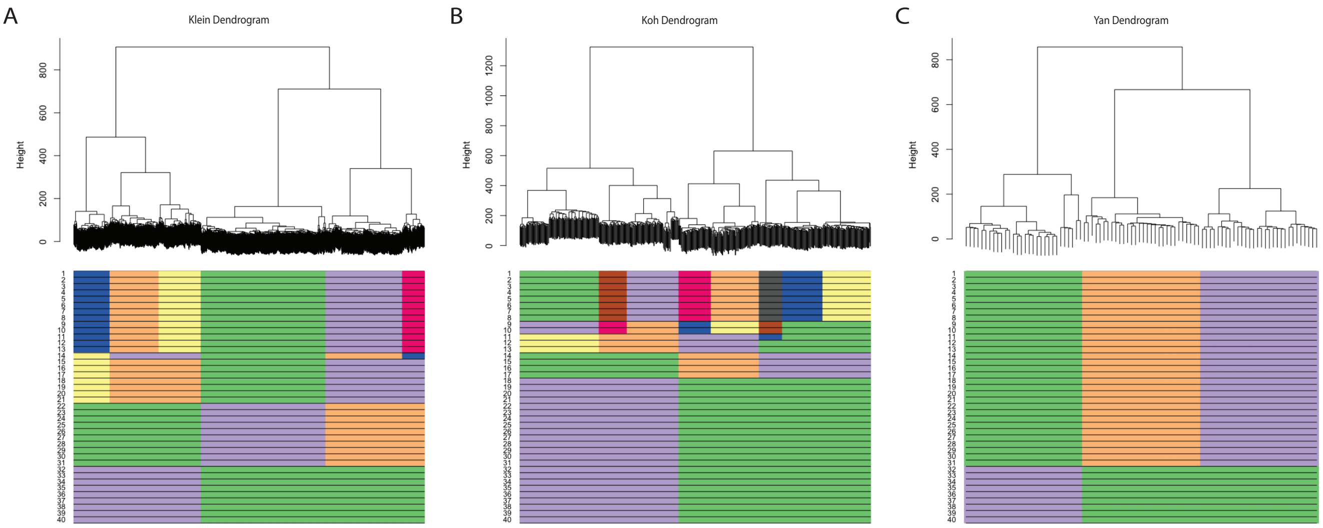

Figure S2. Implementation of the scGPS clustering for a three single cell datasets generated by different sequencing platforms. These datasets are publicly available, and were generated by (Klein et al., 2015) (**A**), (Koh et al., 2016) (**B**), and (Yan et al., 2013) (**C**). The processed datasets by (Soneson and Robinson, 2018) were used for objectively comparing algorithms with scGPS analysis.

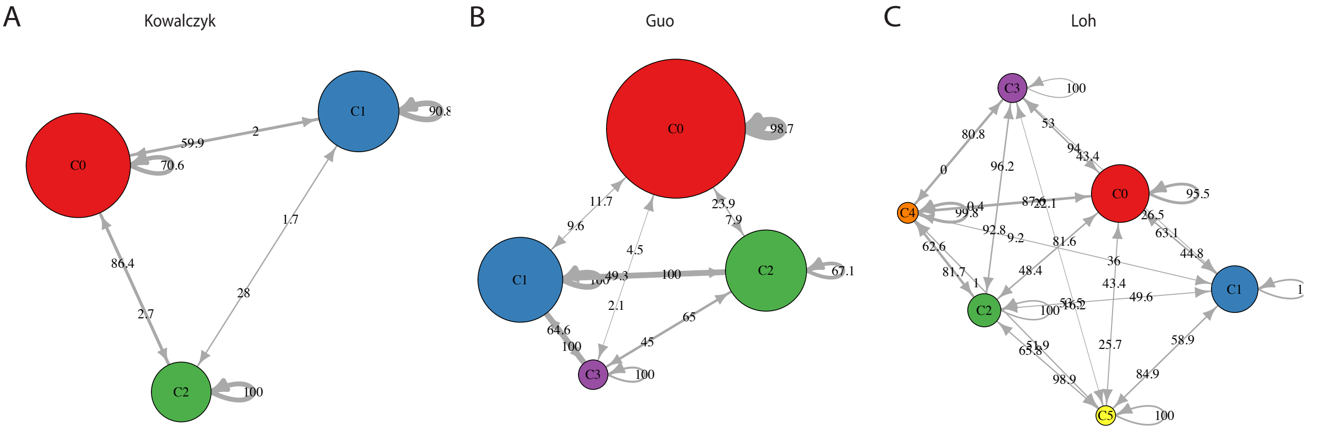

Figure S3. Implementation of the scGPS for trajectory analysis of different single cell datasets. These datasets were made publicly available by (Kowalczyk et al., 2015) (**A**), (Guo et al., 2015) (**B**), and (Loh et al., 2016) (**C**).

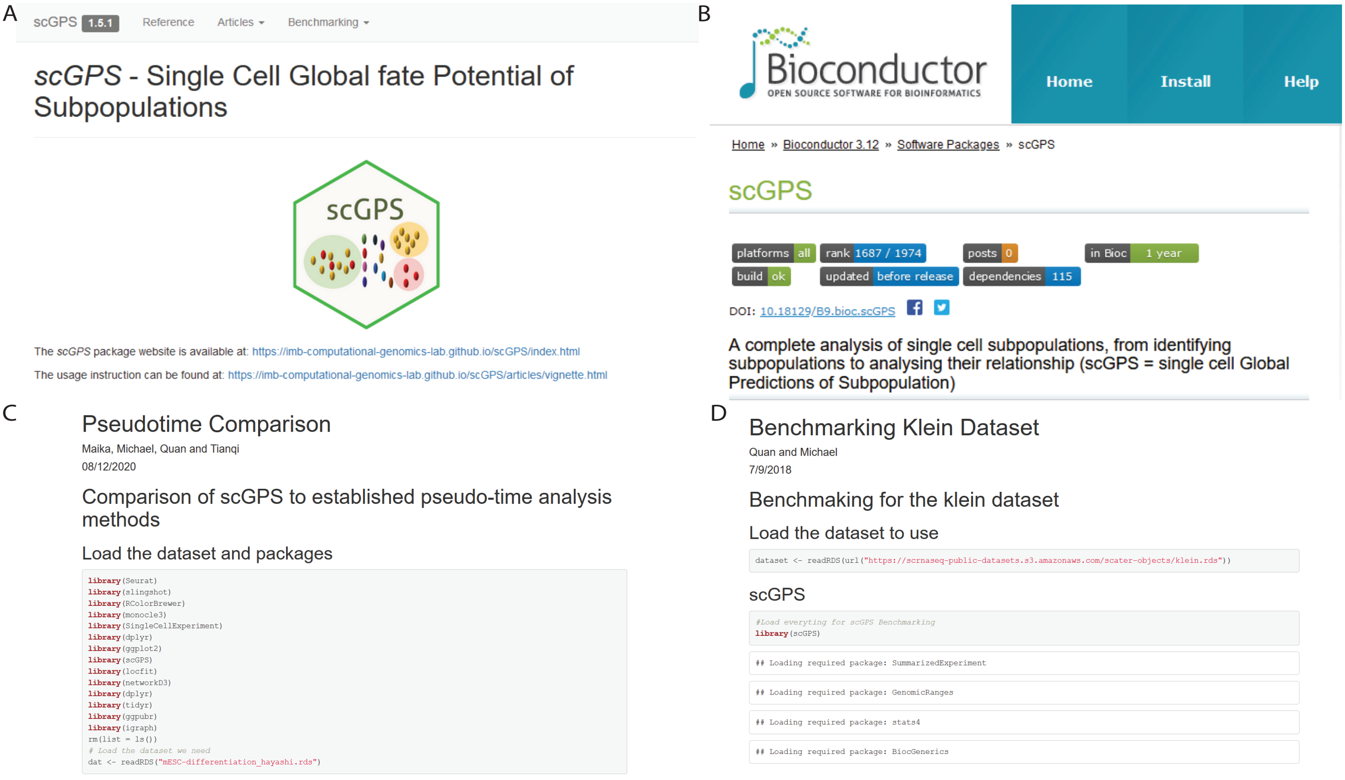

Figure S4. The comprehensive documentation of the scGPS software in Bioconductor and scGPS webpages (publicly accessible at https://github.com/IMB-Computational-Genomics-Lab/scGPS). **A**, scGPS project website. Website contains an overview, guides, function references, benchmarking scripts and outputs. **B**, scGPS Bioconductor webpage. **C**, A reproducible benchmarking documentation for comparing trajectory analysis with Monocle 3 and Slingshot using a number of datasets. **D**, A reproducible benchmarking documentation for comparing clustering analysis with SC3 using various datasets

REFERENCES

Guo, F., Yan, L., Guo, H., Li, L., Hu, B., Zhao, Y., et al. (2015). The transcriptome and dna methylome landscapes of human primordial germ cells. *Cell (Cambridge)* 161, 1437–1452

Klein, A., Mazutis, L., Akartuna, I., Tallapragada, N., Veres, A., Li, V., et al. (2015). Droplet barcoding for single-cell transcriptomics applied to embryonic stem cells. *Cell (Cambridge)* 161, 1187–1201

Koh, P. W., Sinha, R., Barkal, A. A., Morganti, R. M., Chen, A., Weissman, I. L., et al. (2016). An atlas of transcriptional, chromatin accessibility, and surface marker changes in human mesoderm development. *Scientific data* 3, 160109–160109

Kowalczyk, M. S., Tirosh, I., Heckl, D., Rao, T. N., Dixit, A., Haas, B. J., et al. (2015). Single-cell rna-seq reveals changes in cell cycle and differentiation programs upon aging of hematopoietic stem cells. *Genome research* 25, 1860–1872

Loh, K. M., Chen, A., Koh, P. W., Deng, T. Z., Sinha, R., Tsai, J. M., et al. (2016). Mapping the pairwise choices leading from pluripotency to human bone, heart, and other mesoderm cell types. *Cell (Cambridge)* 166, 451–467

Soneson, C. and Robinson, M. D. (2018). Bias, robustness and scalability in single-cell differential expression analysis. *Nature methods* 15, 255–261

Yan, L., Yang, M., Guo, H., Yang, L., Wu, J., Li, R., et al. (2013). Single-cell rna-seq profiling of human preimplantation embryos and embryonic stem cells. *Nature structural & molecular biology* 20, 1131–1139
